# Phages Actively Challenge Niche Communities in Antarctic Soils

**DOI:** 10.1128/mSystems.00234-20

**Published:** 2020-05-05

**Authors:** Oliver K. I. Bezuidt, Pedro Humberto Lebre, Rian Pierneef, Carlos León-Sobrino, Evelien M. Adriaenssens, Don A. Cowan, Yves Van de Peer, Thulani P. Makhalanyane

**Affiliations:** a Centre for Microbial Ecology and Genomics, Department of Biochemistry, Genetics and Microbiology, University of Pretoria, Pretoria, South Africa; b Biotechnology Platform, Agricultural Research Council, Pretoria, South Africa; c Quadram Institute Bioscience, Norwich Research Park, Norwich, United Kingdom; d Department of Plant Biotechnology and Bioinformatics, Ghent University, Ghent, Belgium; e Center for Plant Systems Biology, VIB, Ghent, Belgium; California Department of Water Resources

**Keywords:** Antarctic soils, archaea, bacteria, hypoliths, phages, viromics

## Abstract

By modulating the structure, diversity, and trophic outputs of microbial communities, phages play crucial roles in many biomes. In oligotrophic polar deserts, the effects of katabatic winds, constrained nutrients, and low water availability are known to limit microbial activity. Although phages may substantially govern trophic interactions in cold deserts, relatively little is known regarding the precise ecological mechanisms. Here, we provide the first evidence of widespread antiphage innate immunity in Antarctic environments using metagenomic sequence data from hypolith communities as model systems. In particular, immunity systems such as DISARM and BREX are shown to be dominant systems in these communities. Additionally, we show a direct correlation between the CRISPR-Cas adaptive immunity and the metavirome of hypolith communities, suggesting the existence of dynamic host-phage interactions. In addition to providing the first exploration of immune systems in cold deserts, our results suggest that phages actively challenge niche communities in Antarctic polar deserts. We provide evidence suggesting that the regulatory role played by phages in this system is an important determinant of bacterial host interactions in this environment.

**IMPORTANCE** In Antarctic environments, the combination of both abiotic and biotic stressors results in simple trophic levels dominated by microbiomes. Although the past two decades have revealed substantial insights regarding the diversity and structure of microbiomes, we lack mechanistic insights regarding community interactions and how phages may affect these. By providing the first evidence of widespread antiphage innate immunity, we shed light on phage-host dynamics in Antarctic niche communities. Our analyses reveal several antiphage defense systems, including DISARM and BREX, which appear to dominate in cold desert niche communities. In contrast, our analyses revealed that genes which encode antiphage adaptive immunity were underrepresented in these communities, suggesting lower infection frequencies in cold edaphic environments. We propose that by actively challenging niche communities, phages play crucial roles in the diversification of Antarctic communities.

## Abstract (precis)

In Antarctic environments, the combination of both abiotic and biotic stressors results in simple trophic levels dominated by microbiomes. Although the past two decades have revealed substantial insights regarding the diversity and structure of microbiomes, we lack mechanistic insights regarding community interactions and how phages may affect these. By providing the first evidence of widespread antiphage innate immunity, we shed light on phage-host dynamics in Antarctic niche communities. Our analyses reveal several antiphage defense systems, including DISARM and BREX, which appear to dominate in cold desert niche communities. In contrast, our analyses revealed that genes which encode antiphage adaptive immunity were underrepresented in these communities, suggesting lower infection frequencies in cold edaphic environments. We propose that by actively challenging niche communities, phages play crucial roles in the diversification of Antarctic communities.

## INTRODUCTION

Antarctic terrestrial environments, including open soils, permafrost, and the surface and interior of rocks, are typically oligotrophic and dominated by psychrophilic and psychrotolerant microbial communities (1–4). It has been suggested that the extreme abiotic pressures of the environment, such as temperature, desiccation stress, and UV radiation, are dominant drivers of both the diversity and function of cold-adapted bacterial communities in terrestrial polar deserts (5–7). Similarly, biotic interactions, such as competition, symbioses, horizontal gene transfer (HGT), and predation, have also been shown to play a role in the distribution and diversity of microbial communities in these soil ecosystems (8–10). The presence of viruses, including bacteriophages (henceforth termed phages), in these cold hyper-arid desert soils potentially adds an additional layer of complexity to the microbial system, but the extent to which phage-host interactions play a role in shaping community compositions and processes in cold desert soil niches remains a matter of speculation (11, 12).

Hypoliths, photosynthetic assemblages found below translucent rocks, are widely distributed across hot and cold deserts (13–15). In these arid environments, the absence of plants and larger organisms makes these niche communities, with relatively simple trophic structures, ideal systems for understanding microbial community dynamics (16). There is an increasing body of literature providing detailed insights regarding species richness, composition, and functional diversity of these communities (14, 17–25). These studies have provided clear evidence that hypoliths are essential drivers of functional processes in deserts, including carbon and nitrogen cycling in these ecosystems (26, 27). For instance, a recent study which reconstructed bacterial genomes from Antarctic hypoliths showed that these communities harbor xanthorhodopsin-like proteins and Na-pumping-like rhodopsins (28). Yet, fundamental ecological questions regarding the role played by viruses as drivers of diversification and trophic cycling in hypoliths remain unclear.

Antarctic desert hypolithic communities, in particular, have been shown to contain substantial viral populations, dominated by tailed bacteriophages of the order *Caudovirales* (11, 29–31). Microarray analysis of lithic niches identified an even greater phage diversity, including signatures of RNA bacteriophages of the family *Leviviridae*, ssDNA phage of the family *Microviridae*, and nontailed dsDNA tectiviruses (32). Together, these observations suggest that phages may play an important role in microbial community structures and functions.

The presence of active bacteriophages in a microbial community inevitably leads to the evolution of specialized bacterial defensive measures (33), and a diverse range of bacterial defense mechanisms against parasitic phages have been identified (34, 35). These include adaptive immunity elements, such as the CRISPR-Cas systems, and innate immunity mechanisms, such as restriction-modification (RM) and toxin-antitoxin abortive infection (Abi) systems (34). Recent pangenomic studies have also identified novel defense systems that are widely distributed across bacterial taxa and are thought to play a role in antiphage resistance (36–39). These include the bacteriophage exclusion (BREX) system, encoded by a 4 to 8 gene cluster, that provides resistance to *Siphoviridae* and *Myoviridae* tailed phages by inhibition of phage DNA replication (37), and other less well characterized systems such as the Thoeris, Shedu, and Gabija elements that increase bacterial host resistance to specific groups of phages (38).

Combining the valuable evidence on phage diversity and prevalence in polar desert soils, we hypothesize that phage-host interactions play an important role in shaping the structure of edaphic microbial communities in these environments. To test our hypothesis, we assess the known bacterial defense systems in metagenomic sequence data derived from a niche Antarctic hypolith community. We were able to link a portion of these data to specific phage genomes and propose that phages play an active role in shaping the immunity of Antarctic soil microbial communities.

## RESULTS

### The distribution of antiphage defense mechanisms shows a diversity of innate immunity genes.

**FIG 1:**
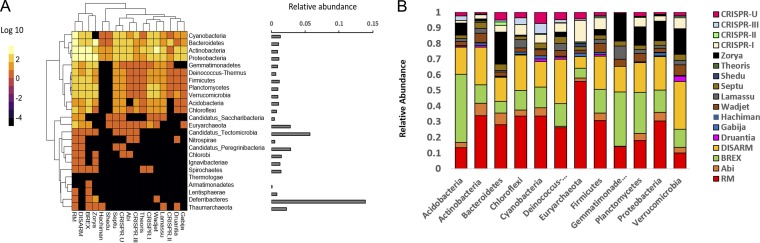
The relationship between relative abundances of taxa and defense systems. (A) The distribution of defense genes across known phyla in the metagenome. The heatmap includes all phyla which represented more than 1% of total scaffolds in the metagenome. The color scaling illustrates absolute abundance values after log_10_-transformation. The bar plot represents the percentage of defense genes detected relative to total gene count for each of the phyla represented in the heatmap. (B) Relative abundance of antiphage defense systems across different phyla. The relative abundance of each defense system is expressed as the ratio between the number of genes for that system and the total number of defense genes for the phylum. The top 12 phylum (representing 99% of contigs in the metagenome) are represented in the barplot.

The distribution of antiphage defense systems in the metagenome was determined by mapping defense genes against the taxonomically assigned contigs. In total, 24,941 defense genes were detected, comprising 1.2% of the entire metagenome gene count. Approximately 40% of these were found in contigs attributed to unknown phyla. The general distribution of defense genes across known phyla was consistent with the relative abundance of each phylum in the metagenome (Fig. 1A, Table S1). *Proteobacteria* harbored the highest number of antiphage genes (5,289 genes, 1.1% of total gene count for this phylum), followed by *Actinobacteria* (3,808, 0.9% of total gene count) and *Bacteroidetes* (2,128, 1.08% of total contig count). RM, DISARM, and BREX systems were the most abundant systems in the metagenome, contributing 67.6% of the total gene hits for antiphage defense systems. On the other side of the spectrum, the defense systems Shedu, Hachiman, and CRISPR-type 2 were present at relatively low abundances, and therefore had little apparent contribution to the global defense system distribution. The average contribution of defense genes to the total gene count per phyla was 1.8%, with *Deferribacteres* and “*Candidatus* Tectomicrobia” as outliers. However, it is important to note that these phyla represent a very small portion of the metagenome, and therefore the possibility that the high percentage of defense genes is biased toward the low gene count for these phyla cannot be disregarded.

10.1128/mSystems.00234-20.6 TABLE S1 Number of total contigs and number of contigs containing antiphage genes in the phyla representing more than 1% of total scaffolds in the metagenome. Download Table S1, DOCX file, 0.01 MB. Copyright © 2020 Bezuidt et al. 2020 Bezuidt et al. This content is distributed under the terms of the Creative Commons Attribution 4.0 International license.

Analysis of the relative contribution of each defense system within each phylum also showed that genes belonging to the RM, DISARM, and BREX systems were the main contributors across the majority of phyla (Fig. 1B). The recently discovered Zorya system was predominantly represented in the phyla *Gemmatimonadetes*, *Bacteroidetes*, *Planctomycetes*, *Proteobacteria*, and *Verrumicrobia*, while CRISPR systems showed the highest contribution in *Cyanobacteria* and *Euryarchaeota*. Interestingly, noncanonical antiphage systems represented more than 50% of the defense systems identified for all phyla aside from *Euryarchaeota*, with *Verrucomicrobia*, *Planctomycetes*, and *Acidobacteria* possessing the highest distribution of noncanonical defense genes.

### Innate immunity is dominated by BREX and DISARM genes.

As highlighted above, antiphage systems across phyla in the hypolith metagenome were dominated by noncanonical innate systems. Further analysis of the distribution of defense genes revealed that antiphage systems in the majority of phyla were dominated by BREX and DISARM genes. The two systems together accounted for 33.4% of defense genes, compared to 31.7% of genes belonging to canonical RM systems.

A total of 3,758 genes for the DISARM system were identified. These included the Class I marker gene *drm*D (449 counts, 11.9% of DISARM genes), which encodes the SNF2-like helicase (39), as well as the Class II marker gene *drm*A (1,020 counts, 17.1% of DISARM genes), which encodes a protein with a putative helicase domain (39). Similarly, a total of 4,598 genes representing all BREX types were identified in the metagenome. Interestingly, the most abundant gene from this system found in the metagenome, *pgl*W (2,640 counts, 57.4% of BREX genes), which codes for a serine/threonine kinase, is specific to the type 2 BREX system, also called the Pgl system (37). By comparison, of the 7,908 RM genes found in the metagenome, the most abundant is *hsdM* (1,423 counts, 18% of RM genes), which encodes a type I DNA methylase responsible for the protection of host DNA (40). In fact, more than 50% of RM defense genes were attributed to type I RM systems.

The third noncanonical system representing more than 10% of the antiphage defense systems in a subset of the phyla, the Zorya system, included a total of 2,411 genes in the metagenome. The majority of these were homologous to the two genes that encode elements of a proton channel, *zor*A and *zor*B. This is a common feature in all types of Zorya system and is thought to cause depolarization of the membrane upon infection (38).

### Type I CRISPR-*cas* genes comprise the bulk of antiphage adaptive immunity genes.

**FIG 2:**
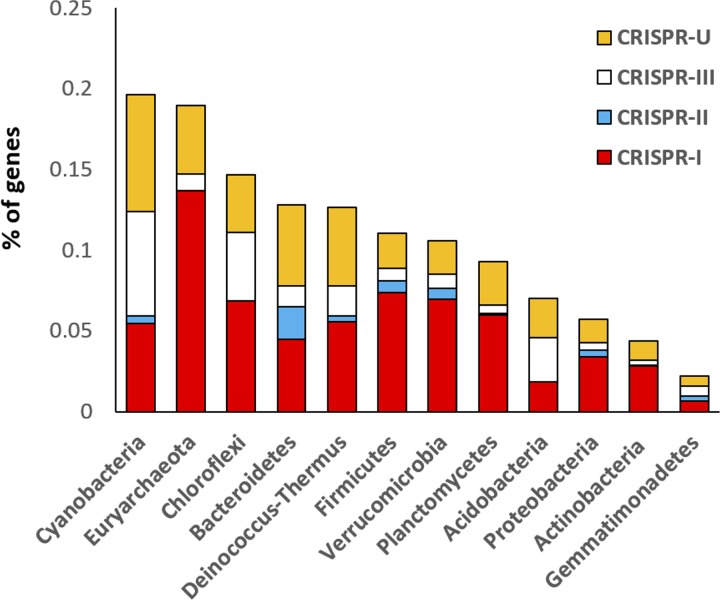
The relative abundances of the three CRISPR-Cas types as well as unclassified defense types across the 12 most dominant phyla. Relative abundances were determined as counts of CRISPR-*cas* genes compared to the total number of genes per phylum.

In total, 2,234 CRISPR-*cas* genes were identified in 1,601 contigs by searching for shared sequence similarities against the conserved domains database (CDD). A substantial proportion of all classified CRISPR-*cas* loci (71.4%) belonged to type I CRISPR-Cas systems, followed by type III (18.5%) and type II (10.2%) (Table S2). While the abundance of *cas* I-B loci sequences in the public databases suggests that the Cas-I mechanism is the most common in both bacteria and archaea (20% and 30% of total CRISPR loci, respectively) (41), less than 3% of these loci were present in our composite metagenome (Fig. 2, Table S2). Surprisingly, CRISPR-*cas* loci linked to Types I-C and I-E were the most prevalent, at 24.1% and 12.9% of classified CRISPR-*cas* loci, respectively. Another subtype identified at higher relative abundances than previously reported (41) was I-U, at 10.76% of classified *cas* loci. This subtype is characterized by the marker GSU0054 domain, which was the fourth most abundant *cas* CDD overall (108 occurrences) after *cas4*, *cas1*, and *cas2*.

10.1128/mSystems.00234-20.7 TABLE S2 Marker genes used for screening and identification of the main types of antiphage defense systems in the hypolith metagenome. Download Table S2, DOCX file, 0.01 MB. Copyright © 2020 Bezuidt et al. 2020 Bezuidt et al. This content is distributed under the terms of the Creative Commons Attribution 4.0 International license.

### Phage presence in the niche community is correlated with the CRISPR arrays.

CRISPR arrays represent the history of infection by invading DNA (e.g., phages and plasmids) (42, 43), and a study of their composition and frequencies provides insights into phage-host interactions in an ecological context (44). A total of 878 CRISPR arrays harboring 10,292 spacers were identified in the metagenome, with an average length of 36 protospacers per array (Fig. S1A). CRISPR array sizes ranged from 2 to 249, with the majority (83.5% of total arrays) falling between 2 and 18 protospacers per array (Fig. S1B).

**FIG 3:**
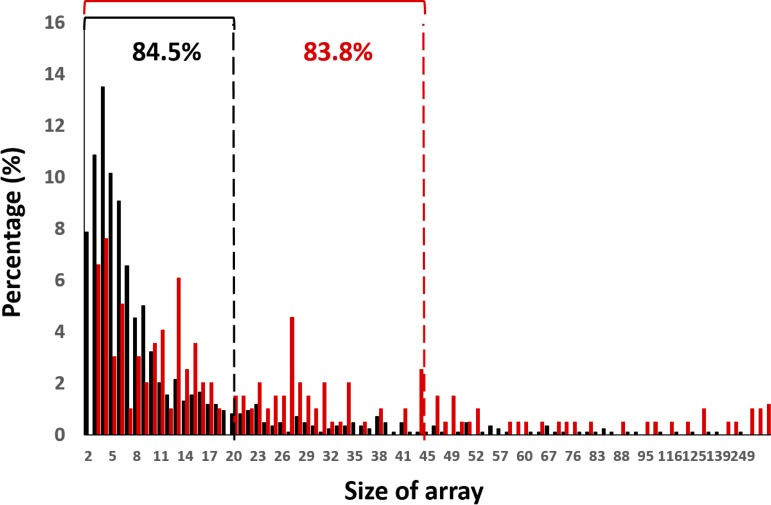
The distribution of CRISPR array sizes in the hypolith metagenome and a groundwater metagenome. Spacers for the hypolith and groundwater metagenomes are colored as red and black, respectively. Array size distributions in each metagenome are expressed as percentage of total number of arrays detected for each metagenome. Cutoffs below 85% are indicated by black and red dashed lines for both the hypolith and groundwater metagenomes, respectively, and represent the size range containing the majority (≤85%) of the array population.

The distribution of CRISPR array sizes in the metagenome was compared to data collected from a groundwater microbiome (45) to compare the array size distributions from environments with potentially different phage-host dynamics (11). The results show that CRISPR arrays in the hypolith metagenome exhibited a smaller and narrower size range compared to the groundwater community metagenome (Fig. 3). This suggests the existence of distinct phage infection frequencies between the different environments, i.e., with lower infection frequencies in the cold edaphic community.

10.1128/mSystems.00234-20.1 FIG S1 Protospacers and their respective frequency per arrays. (A) The distribution of protospacers arranged according by length. (B) The distribution of arrays according to frequency of protospacers per array. Download FIG S1, DOCX file, 0.06 MB. Copyright © 2020 Bezuidt et al. 2020 Bezuidt et al. This content is distributed under the terms of the Creative Commons Attribution 4.0 International license.

**FIG 4:**
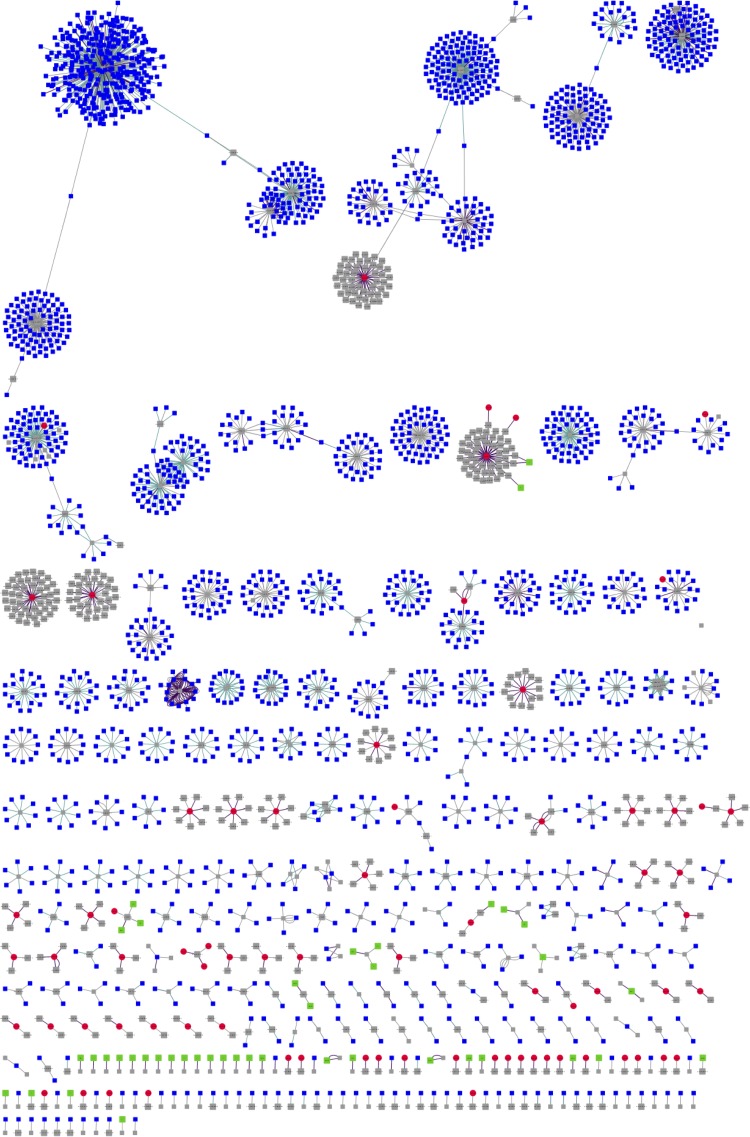
Network visualization of the relationship between the hypolith virome, CRISPR-spacers, and reference data sets. The nodes in the network are CRISP-R spacers (gray), hypolith metavirome (red), IMG/VR viromes (blue), and Antarctic soil viromes (green). Positive alignments between data sets are represented by the connections (edges) between nodes, with the connection distance being inversely proportional to the alignment strength.

In addition to using the CRISPR array as a tool for understanding infection history, the viral population in the Antarctic soil community was also assessed by assembly of the metavirome. A total of 793 contigs was assembled from the metagenomic sequence data using VirSorter (46). Taxonomic annotation of these contigs, using a database of viral reference genomes (47), unambiguously assigned 645 of these as viral, 560 of which were further assigned to the order of tailed phages, the *Caudovirales*. Within this order, the majority of contigs were assigned to *Siphoviridae* (52%), followed by unclassified *Caudovirales* (14%), and viruses with no assigned family (13%) (Fig. S2). To access the correlation between the viral contigs and the CRISPR arrays, spacers from the metagenome were matched to both the VirSorter contigs and a set of contigs from environmental data sets (IMG/VR) (48), which allowed for the taxonomic assignment of 394 (3.8% of total number of spacers) CRISPR-*cas* spacers (Fig. S3). The resulting similarity network (Fig. 4) showed that all 73 VirSorter phage contigs included in the network (red nodes) matched to CRISPR-*cas* spacers (gray nodes). This result suggests that a substantial fraction (11.3%) of the identified viral population had a history of infection *in situ* in the host population, and may therefore be actively involved in shaping the adaptive immunity of the microbial community. In addition, several distinct clusters showed matches between a single VirSorter contig and several spacers, suggesting these viral contigs are common infection agents.

10.1128/mSystems.00234-20.2 FIG S2 Metavirome contig annotations. Percentages of families for the annotated are represented in the pie chart, and the more detailed taxonomy annotation of the contigs is shown in the dendrogram. The number of contigs with the same annotation is represented by the bar plot. Download FIG S2, DOCX file, 0.1 MB. Copyright © 2020 Bezuidt et al. 2020 Bezuidt et al. This content is distributed under the terms of the Creative Commons Attribution 4.0 International license.

10.1128/mSystems.00234-20.3 FIG S3 Protospacers detected in the metagenomes and viral contigs. (A) Taxonomic distribution of the protospacers detected in the hypolith metagenome. (B) Taxonomic distribution of the viral contigs assembled using VirSorter. Fractions of the most abundant taxa are expressed in percentages, which were calculated as a percentage of the total number of protospacers and contigs, respectively. Download FIG S3, DOCX file, 0.2 MB. Copyright © 2020 Bezuidt et al. 2020 Bezuidt et al. This content is distributed under the terms of the Creative Commons Attribution 4.0 International license.

**FIG 5:**
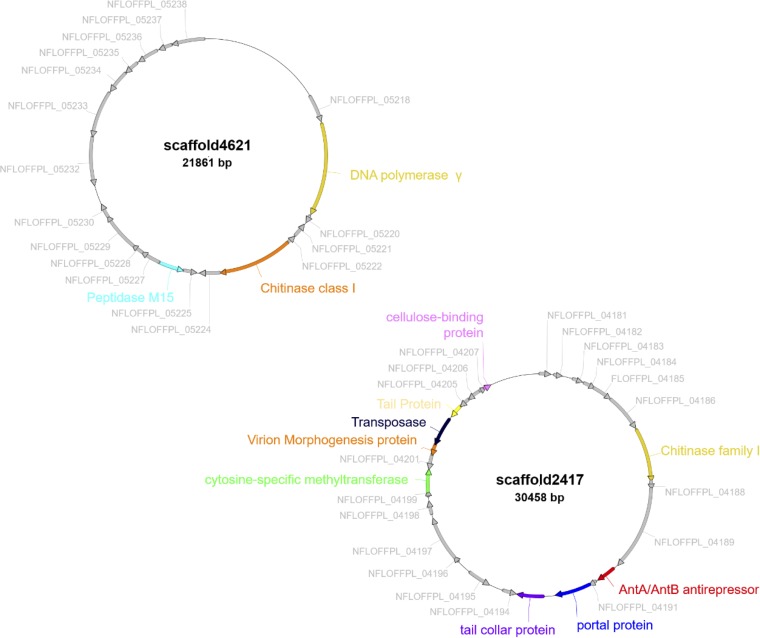
Gene architecture of the VirSorter contigs with matches to multiple CRISPR array spacers. These contigs were selected based on the presence of virulence and immunity evasion genes. Genes with known functions are highlighted to distinguish from hypothetical genes (gray).

Functional analysis using eggNOG showed the presence of genes that facilitate infection, such as genes that code for chitinases, which are involved in the degradation of the protective biofilm (49), as well as an AntA/AntB antirepressor gene, thought to be involved in phage anti-immunity (50) (Fig. 5). In addition, the eggNOG functional analysis of the 645 VirSorter viral contigs also revealed the presence of genes contributing to phage virulence (Table 1), the most abundant of which encode methyltransferases, which are actively involved in the evasion of the RM systems (51). This result suggests the possibility of an evolutionary pressure for the phages to develop evasion mechanisms against their hosts, which further hints at active phage-host dynamics in these long-enduring Antarctic hypoliths.

**TABLE 1:** The number of genes linked to the contigs^*a*^

eggNOG annotations	No. of genes
DNA methylase	50
Adenine-specific methyltransferase	28
Cytosine-specific DNA methylase	11
Chitinase class I	6
Virulence-associated protein E	2
Repressor protein	1
Antirestriction ArdA family protein	1
Type 12 methyltransferase	1
Virulence-associated protein	1
Methyltransferase, type 11	1
Methyltransferase FkbM family	1

a eggNOG was used for VirSorter contig annotations, which were then linked to the virulence phenotypes. The number of genes for each category is shown in descending order.

## DISCUSSION

Due to the relatively simple trophic structures in cold desert systems, including Antarctic soils, cryptic microbial communities are considered to be important drivers of local ecosystem services (52). However, the extent to which these communities are influenced by phages remains largely unexplored. Such interactions may shape the diversification and community interactions in cold desert systems. Qualitative surveys of Antarctic metaviromics have reported a high diversity of viruses associated with microbial communities of open soils, and cryptic niches (12, 29). Evidence, albeit limited, that Antarctic soil phages exist predominantly in a lysogenic rather than lytic lifestyle (30) has led to suggestions that the functional role of phages in this spatially restricted, water-constrained desert soil niche may be limited (11).

The results presented in this study provide the first evidence of interaction between phage and hosts in this psychrophilic edaphic environment. This is most evident in the correlation between the metavirome of the hypolith community and the CRISPR-arrays, which suggest the active evolution of the adaptive immune system against local viral threats. This idea of community adaption to local phage threat is further implied by the positive correlation between the CRISPR arrays and viruses extracted from local soils. In fact, a previous study (19) has already suggested that recruitment from surrounding soils plays an important role in the development of hypoliths, and this might also be extended to the recruitment of phages from the surrounding ecosystem. Another indication of active interaction between phage and host is suggested by the presence of several methyltransferases in the metagenome-assembled viral contigs, which are a hallmark of viral evasion against native host RM systems (47, 51). Other genes found in this set of virome contigs includes genes specifically involved in the degradation of biofilm matrices and evasion against RM systems, further suggesting a complex network of interactions at play between phages and their hosts in the hypolithic environment.

While the metagenomics data analyzed in this study does not give a direct indication of the temporal scale of the phage-host interactions occurring in the hypolith, the short sizes of CRISPR-array sizes in the hypolith metagenome suggest a low frequency of infection. This low frequency is further hinted at when comparing the hypolith CRISPR-array sizes with those of a more fluid and homogenous environment, where viral-host interactions are assumed to be a frequent occurrence (45). Together, these results imply a model for viral-host interactions in hypoliths that follows the “static-step-static” development model suggested by Pointing et al. (53), driven by the stochastic and intermittent nature of rain events in such water-limited ecosystems. A surprising result from this study is the prevalence of noncanonical innate immunity systems, the most prominent of which are the BREX and DISARM systems. While these two systems have been shown to be widespread in bacteria using a pangenomic data set (37, 39), the present study represents the first evidence for the prevalence of these systems in ecological samples. As such, this result implies that noncanonical innate immunity is more important for antiphage microbial community defense than previously thought, and should therefore be the focus for future studies into innate immunity in the ecological context. There are also indications from the hypolith metagenome that the prevalence of noncanonical innate immunity over traditional RM and Abi systems for defense against phages is related to the adaptation of the hypolith communities to specific local viral populations. For instance, the Zorya system, the third most prevalent noncanonical immunity system in the metagenome, is hypothesized to operate similarly to the Abi system (38). In turn, Zorya systems provide resistance against a limited range of phages, including the ssDNA family *Microviridae* (38), which has been shown to be prevalent in Antarctic aquatic and soil niches (54).

**Conclusion.** Together, these results are not consistent with the suggestion that the constraints of the environment, such as low temperatures, low a_w_, and resulting limited capacity for interparticle diffusion, lead to extremely localized phage-host interactions (11). Rather, the data are suggestive of a dynamic and continual interaction between host and phage. Nevertheless, interparticle communication and exchange may be limited to brief periods when bulk liquid water is present, such as after snow melt, for example. Furthermore, the low metabolic rates (the inevitable consequence of Arrhenius effects [temperature dependence of reaction rates] in cold environments) should also limit the rates at which phages can replicate and propagate, further limiting the frequency of interactions with their hosts (55). We suggest that the localized nature of host-phage interactions in the hypolith niche and the limited interparticle communication, where bacterial hosts are not frequently challenged by novel phage threats, leads to a reliance of microbial communities on innate immunity as the primary defense against phage infection. The smaller sizes of CRISPR arrays in the Antarctic soil metagenome sequences compared to those from a temperate aquatic environment, and the underrepresentation of CRISPR systems, give further credence to the temporally sporadic interaction between phages and their hosts. Nevertheless, the correlation between the metavirome and the CRIPR-*cas* arrays, together with the presence of bacteriophage evasion genes in the metavirome, suggest that phage-host interactions within the hypolith community are a dynamic process that leads to coevolution of both phages and hosts. We therefore suggest that phages play a hitherto underestimated role in driving the evolution of Antarctic soil microbial communities by shaping their collective immunity.

## MATERIALS AND METHODS

### Sample collection, DNA extraction, and metagenomic sequencing.

The sample collection, DNA extraction, and metagenomic sequencing protocols used in this study have been described previously (28). Briefly, a total of 50 samples were collected from hypolithic niches in the Antarctica Miers Valley (GPS 78°09’36.0”S 164°06’00.0”E) and stored in sterile Whirl-Pak bags (Nasco International, Fort Atkinson, WI, USA) at –20°C. Metagenomic DNA was extracted from each sample using a Power Soil DNA isolation kit (MO BIO, Carlsbad, CA, USA), and the purified DNA was pooled before further processing. Purified DNA was sheared into fragments of approximately 300 bp and further purified from 1% agarose gels. Subsequent sequencing was performed using Illumina HiSeq-2000 paired-end technology (2 × 101 bp), and the resulting reads were trimmed and assembled as described below.

### Metagenome assembly and taxonomical annotation.

Metagenomic DNA sequence data were quality filtered by trimmomatic version 0.36 using a phred cutoff of >30 (56). The assembly of high-quality reads from the metagenome sequence data set was conducted using the IDBA-UD tool (57) and contig lengths were extended (scaffolded) using SSPACE Basic (57). The statistics for the assembly of the metagenome are presented in Table S3. We estimated diversity and coverage using Nonpareil v3.0 (see Fig. S4) (58). To determine the relative abundances of bacteria, archaea, and viruses, reads were taxonomically assigned using kaiju v1.7.3 (Fig. S5) (59). Contigs were taxonomically assigned using the MEGAN v6 pipeline (60) with the NCBI taxonomy database for taxon ID assignment.

10.1128/mSystems.00234-20.4 FIG S4 Summary statistics for metagenomic data. Coverage and diversity estimates are shown as sequencing effort (bp) versus estimated average coverage. The curve shows that hypolithic metagenomes had a coverage in excess of 95% and that diversity was estimated at 19.4%. Download FIG S4, DOCX file, 0.10 MB. Copyright © 2020 Bezuidt et al. 2020 Bezuidt et al. This content is distributed under the terms of the Creative Commons Attribution 4.0 International license.

10.1128/mSystems.00234-20.5 FIG S5 The relative abundance of microbial taxonomic groups ranked from highest to lowest. The bar plot shows the ten most abundant phyla found in the hypolith dataset. Download FIG S5, DOCX file, 0.1 MB. Copyright © 2020 Bezuidt et al. 2020 Bezuidt et al. This content is distributed under the terms of the Creative Commons Attribution 4.0 International license.

10.1128/mSystems.00234-20.8 TABLE S3 Quality and summary statistics for the hypolith metagenome. Assembly was done using IDBA-UD. Download Table S3, DOCX file, 0.01 MB. Copyright © 2020 Bezuidt et al. 2020 Bezuidt et al. This content is distributed under the terms of the Creative Commons Attribution 4.0 International license.

### Detection of the innate and adaptive defense systems.

Metagenomic contigs were used for functional gene predictions using prodigal v2.50, with the –meta parameter implementation (61). Predicted genes were subsequently screened for domain similarity with known defense systems against the conserved domain database (CDD) of clusters of orthologous groups (COGs) and protein families (Pfams) using RPS-BLAST (E value <1e-02) (49). These results were manually filtered for the identification of phage-specific defense systems, which include restriction-modification (RM), bacteriophage exclusion (BREX), abortive infection (Abi), defense island system associated with restriction-modification (DISARM), and other recently identified systems using a refined list of COG and Pfam position-specific score matrices (PSSMs) for marker genes in these systems (37–39, 62). A list of the marker genes used in this study can be found in Table S2. Defense genes that could not be clustered into specific systems were classified as ambiguous and were not considered for subsequent analysis.

Open reading frames (ORFs) predicted using prodigal v2.50 were queried against the CDD database for the presence of putative CRISPR-*cas* genes (63), using DELTA-BLAST at a cutoff E value of 1e-03. Multigene *cas* modules were identified as those having multiple *cas* annotated genes with ≤5 ORF spacings. Type and subtype classifications were assigned following the updated classification set by Makarova et al. (41).

### Phage genome identification and CRISPR spacer matching.

Antarctic hypolith phage genomes were identified from the assembled metagenome using VirSorter (46) on the iVirus platform hosted by Cyverse (64), using the virome database and the microbial decontamination option. Only predictions of categories 1, 2, 4, and 5 were used (phages and prophages identified with the “pretty sure” and “quite sure” qualification). Additional phage environmental phage contigs were downloaded from the IMG/VR database version 2018-07-01_4 (48) and used for the network construction. Taxonomic assignment of assembled contigs was performed by using the DIAMOND blastx function with a viral database downloaded from the NCBI Viral Genomes Resource and E value set to 1e-5. ORFs of VirSorter contigs were predicted using Prodigal v2.50 (47, 65) with the virus genomes setting and annotated using eggNOG-mapper v1 (66) with the DIAMOND option and the EggNOG v4.5.1 database (67). Annotations were visualized with the ApE v2.0.55 plasmid editor (http://jorgensen.biology.utah.edu/wayned/ape/).

The CRISPR recognition tool (CRT) v1.2 was used with the default settings to search for CRISPR arrays in the hypolith metagenome (68). The identified spacers in the arrays were matched with the VirSorter phage database and the IMG/VR database using blastn of the BLAST+ suite with the following parameters: -qcov_hsp_perc 80 -task blastn -dust no -soft_masking false (69). Spacer matches of >90% sequence identity for the VirSorter genomes and >95% identity for the IMG/VR genomes were exported and visualized as a network in Cytoscape (70), where the nodes are spacers (gray) or genomes (blue = IMG/VR; red = VirSorter) and the edges blastn matches.

### Sequence data availability.

All sequence data linked to this study have been deposited in the NCBI SRA under accession number SRR3471615.
