# Supplementary material for: Phages Actively Challenge Niche Communities in Antarctic Soils
Source: mSystems. 2020 May 5;5(3):e00234-20. doi: 10.1128/mSystems.00234-20 (PMC7205518; doi:10.1128/mSystems.00234-20)
Supplement: TABLE S1 [file mSystems.00234-20-st001.docx]

**Table S1.** Number of total contigs and number of contigs containing anti-phage genes in the phyla representing more than 1% of total scaffolds in the metagenome.

| **Phyla** | **Defence gene contigs** | **Total contigs** |
| --- | --- | --- |
| Acidobacteria | 448 | 9722 |
| Actinobacteria | 8014 | 155967 |
| Bacteroidetes | 3362 | 95031 |
| Candidatus_Tectomicrobia | 158 | 5407 |
| Chloroflexi | 504 | 13793 |
| Cyanobacteria | 1767 | 28281 |
| Deinococcus-Thermus | 427 | 12795 |
| Euryarchaeota | 116 | 1602 |
| Firmicutes | 506 | 9550 |
| Gemmatimonadetes | 495 | 11467 |
| Planctomycetes | 1284 | 40716 |
| Proteobacteria | 9785 | 195338 |
| Verrucomicrobia | 996 | 24301 |
