# Supplementary material for: Phages Actively Challenge Niche Communities in Antarctic Soils
Source: mSystems. 2020 May 5;5(3):e00234-20. doi: 10.1128/mSystems.00234-20 (PMC7205518; doi:10.1128/mSystems.00234-20)
Supplement: TABLE S2 [file mSystems.00234-20-st002.docx]

**Table S2.** Marker genes used for screening and identification of the main types of anti-phage defense systems in the hypolith metagenome.

| **System** | **Marker Genes** |
| --- | --- |
| **R.M** | *hsdR*, *hsdM/hsdS*, *yhdJ, ssl2, mcrA, yeeA , mrr, COG2810, mcrBC* |
| **DISARM** | *drmA, drmB, drmC , drmD*, *drmMI, drmMII* |
| **BREX** | *brxA, brxC*, *brxHI, brxL, brxP, pglX*, *pglW*, *pglZ* |
| **Druantia** | *druE,* *druM* |
| **Abi** | *abiEi*, abiEii |
| **Zorya** | *zorA*/*zorB, zorC,* zorD, zorE |
| **Septu** | *ptuA*, *ptuB* |
| **Gabija** | *gajA,* *gajB* |
| **Theoris** | *thsA, thsB* |
| **CRISPR-cas Type I** | *cas3 , cas5, csp1, csp2* |
| **Type I-A** | *cas8a1, csx13* |
| **Type I-B** | *cas1-HMARI*,  *cas1-MYXAN, csh2, cst2, cmx8* |
| **Type I-C** | *cas5d, csd1, csx17* |
| **Type I-D** | *csc1, csc2, csc3, cas10d, cas8c* |
| **Type I-E** | *cse1, cse2, cas5e, PRK13921* |
| **Type I-F** | *csy1, csy2, csy3, csy4, cas1-YPEST* |
| **Type I-U** | *GSU0052, GSU0053, GSU0054, csb1, csb2, csb3, csx15* |
| **CRISPR-cas Type II** | *csn1, cas-NMENI, cas9* |
| **Type II-B** | *csx12* |
| **CRISPR-cas Type III** | *cas10, cas6, csx1, csx3* |
| **Type III-A** | *csm1, csm2, csm4, csm5, csm6, TM1806* |
| **Type III-B** | *cmr1, cmr3, cmr4, cmr5, cmr6, csx1* |
| **Type III-BC** | *cmr1, cmr3, cmr4, cmr5, cmr6, csx1* |
| **Type III-D** | *csx10* |
