# Supplementary figures and images for: Phages Actively Challenge Niche Communities in Antarctic Soils
Source: mSystems. 2020 May 5;5(3):e00234-20. doi: 10.1128/mSystems.00234-20 (PMC7205518; doi:10.1128/mSystems.00234-20)

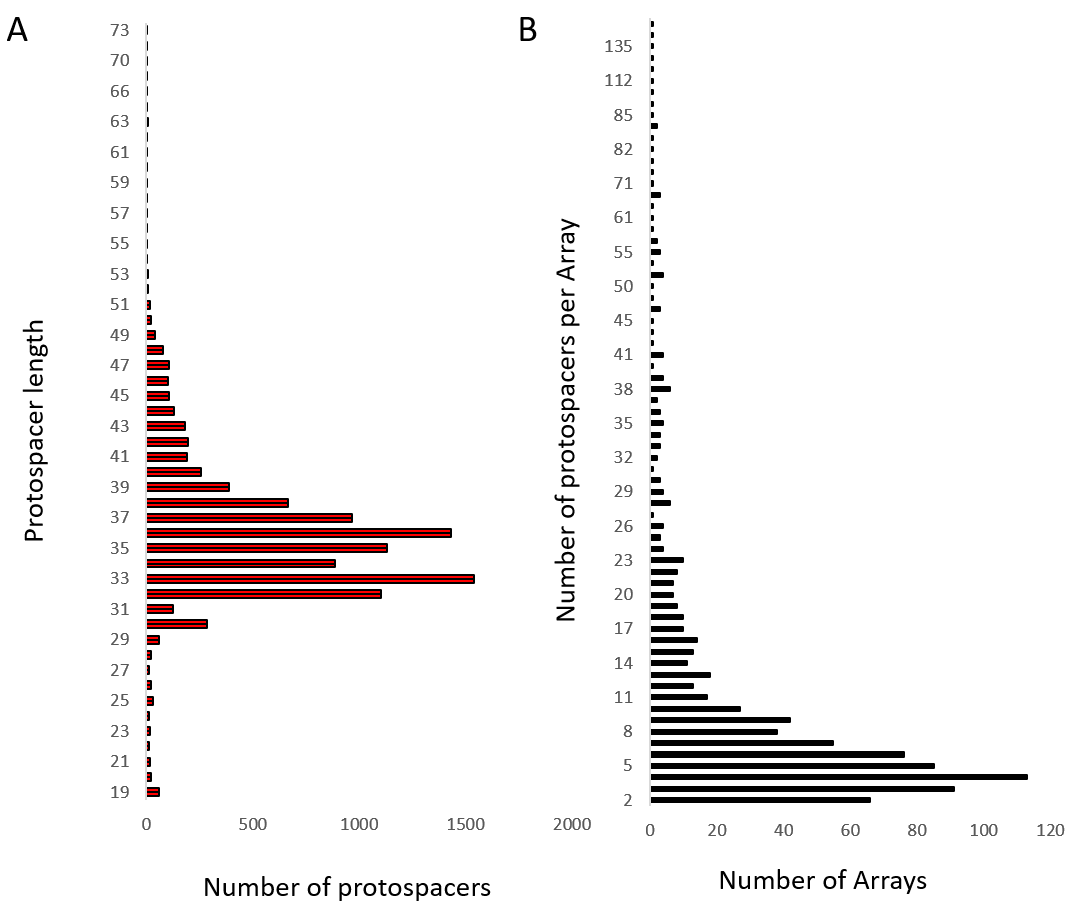

Supplement: FIG S1 [file mSystems.00234-20-sf001.docx]

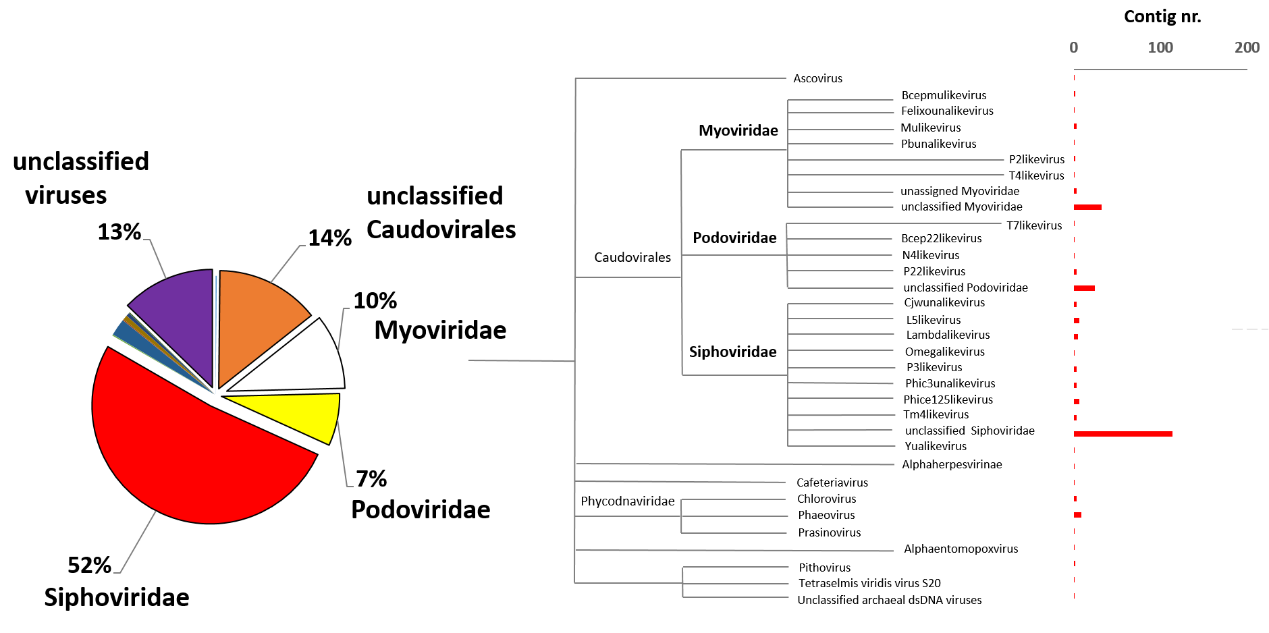

Supplement: FIG S2 [file mSystems.00234-20-sf002.docx]

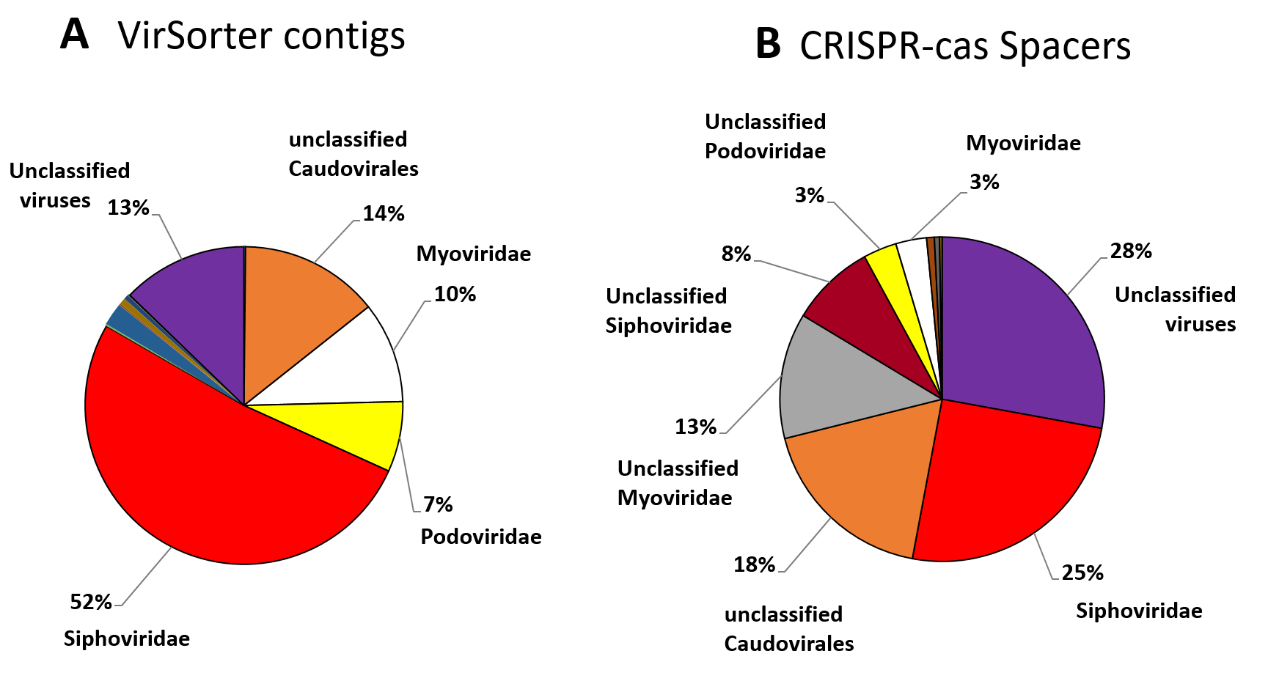

Supplement: FIG S3 [file mSystems.00234-20-sf003.docx]
