# Supplementary material for: Phages Actively Challenge Niche Communities in Antarctic Soils
Source: mSystems. 2020 May 5;5(3):e00234-20. doi: 10.1128/mSystems.00234-20 (PMC7205518; doi:10.1128/mSystems.00234-20)
Supplement: TABLE S3 [file mSystems.00234-20-st003.docx]

**Table S3**. Quality, and summary statistics for the hypolith metagenome. Assembly was done using IDBA-UD.

|  | **IDBA-UD** |
| --- | --- |
| Total contigs | 936,223 |
| GC (%) | 62.08 |
| N50 | 4145 |
| L50 | 55490 |
| contigs (>= 1000 bp) | 288458 |
| contigs (>= 10000 bp) | 18792 |
| Contigs (>=50000 bp) | 1257 |
