# Supplementary material for: Phages Actively Challenge Niche Communities in Antarctic Soils
Source: mSystems. 2020 May 5;5(3):e00234-20. doi: 10.1128/mSystems.00234-20 (PMC7205518; doi:10.1128/mSystems.00234-20)
Supplement: Supplementary file 5 [file mSystems.00234-20-sf004.docx]

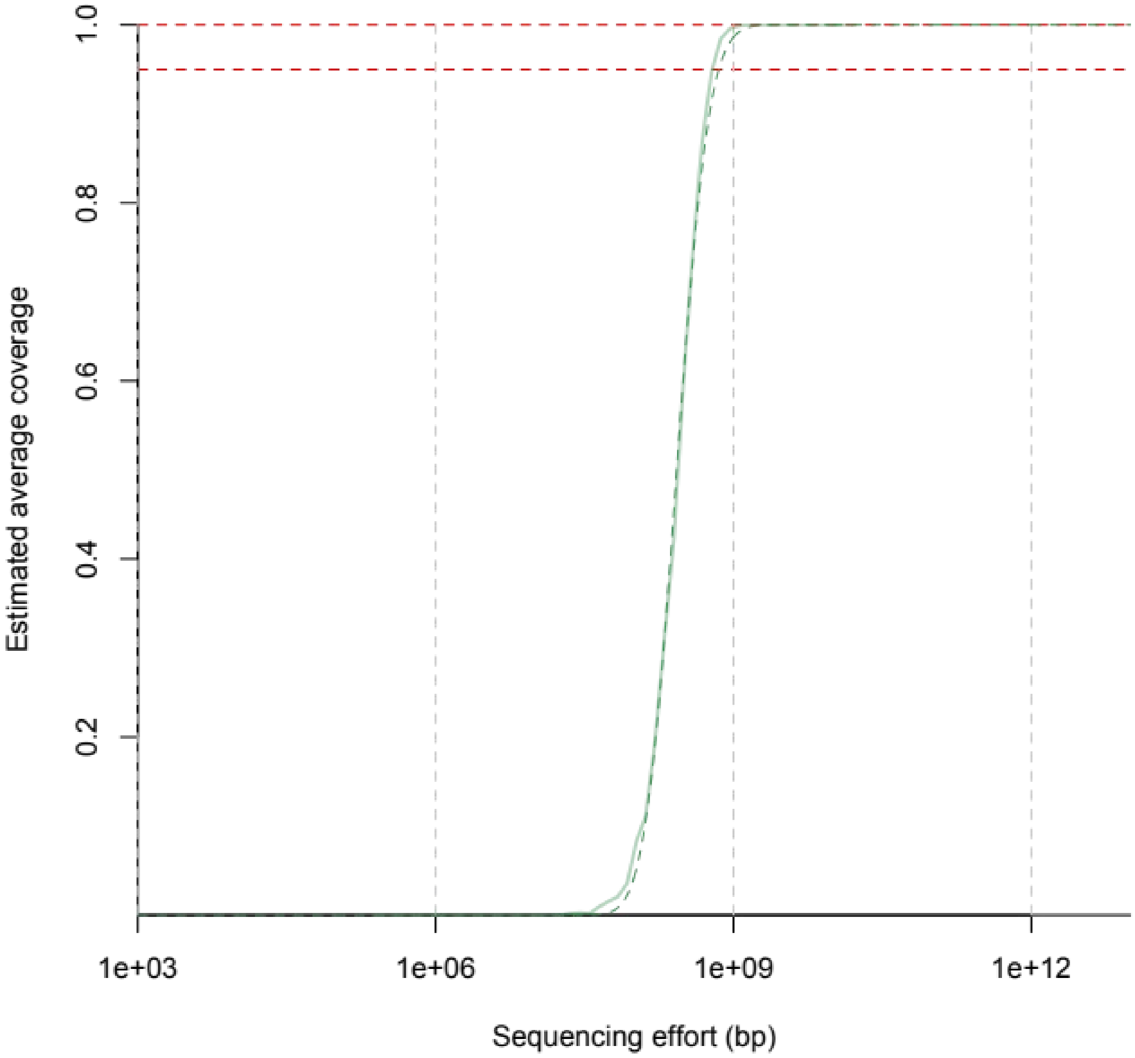

Estimated average coverage
1.0
0.8
0.6
0.4
0.2
1e+03
1e+06
1e+09
1e+12
Sequencing effort (bp)
