# Supplementary material for: Phages Actively Challenge Niche Communities in Antarctic Soils
Source: mSystems. 2020 May 5;5(3):e00234-20. doi: 10.1128/mSystems.00234-20 (PMC7205518; doi:10.1128/mSystems.00234-20)
Supplement: Supplementary file 6 [file mSystems.00234-20-sf005.docx]

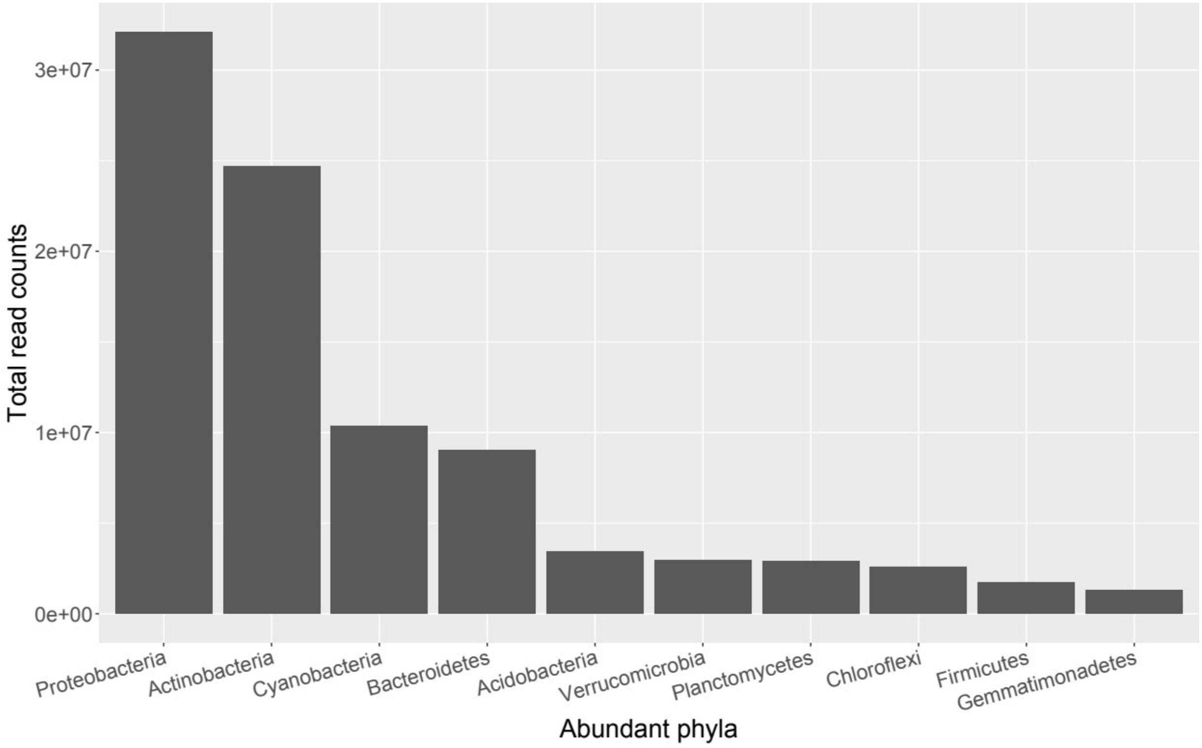

3e+07
2e+07
1e+07
0e+00
Total read counts
Proteobacteria
Actinobacteria
Cyanobacteria
Bacteroidetes
Acidobacteria
Verrucomicrobia
Planctomycetes
Chloroflexi
Firmicutes
Gemmatimonadetes
Abundant phyla
